# Monitoring flood risk evolution: A systematic review

**DOI:** 10.1016/j.isci.2024.110653

**Published:** 2024-08-23

**Authors:** Nele Rindsfüser, Andreas Paul Zischg, Margreth Keiler

**Affiliations:** 1 Oeschger Centre for Climate Change Research, Mobiliar Lab for Natural Risks, University of Bern, Bern, Switzerland; 2 Institute of Geography, University of Bern, Bern, Switzerland; 3 Department of Geography, University of Innsbruck, Innsbruck, Austria; 4 Institute for Interdisciplinary Mountain Research, Austrian Academy of Sciences, Innsbruck, Austria

**Keywords:** natural sciences, Earth sciences, environmental policy

## Abstract

Land-use change, climate change, human interventions, and socio-economic developments influence the evolution of the risk components hazard, exposure, and vulnerability, and consequently of flood risk. Adaptive flood risk management is a way to cope with evolving risks, but it requires measuring the evolution of risks. To develop principles of flood risk monitoring, we systematically reviewed scientific literature on flood risk evolution analyses. The reviewed publications indicate a wide spread in increase or decrease of flood risk evolution over decades. Furthermore, the publications show a high diversity in factors and methods for flood risk evolution analysis and indicate the main challenges for developing flood risk monitoring. Flood risk monitoring needs the systematic detection of flood risk evolution by periodically (re)evaluate the factors that influence the risk components—hazard, exposure and vulnerability—modeling those risk components and combining them to quantify flood risk.

## Graphical abstract

**Figure:**
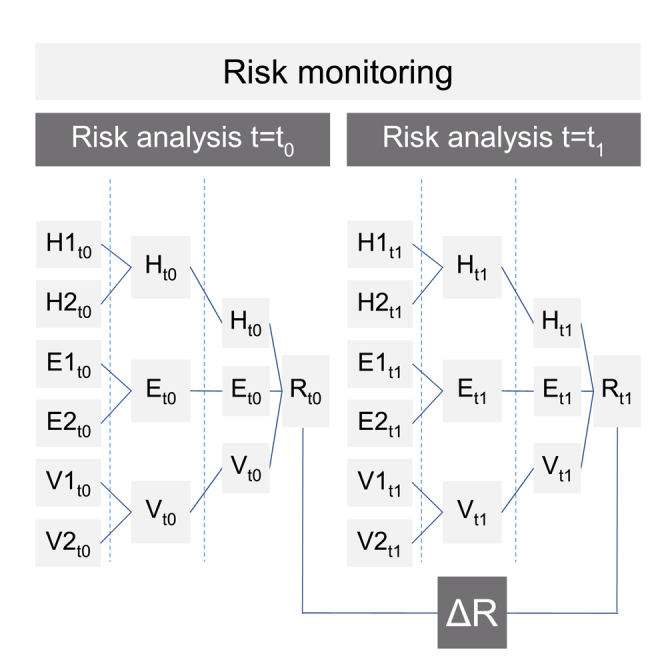

## Introduction

On a global level, floods have caused the most reported disasters or loss events related to weather, climate, and water hazards (44%), led to the second highest economic losses (31%) and the third highest number of deaths (16%) from 1970-2019.^1^ In a general context, flood risk can be expressed in different dimensions, e.g., as the potential future losses due to floods or as the probability of an adverse outcome that arises from the combination of a natural hazard and vulnerable elements within a community.^2^ The analysis and assessment of flood risk is an interdisciplinary approach and may differ depending on the foci and discipline.^3^

Following the general concept of the Intergovernmental Panel on Climate Change IPCC^4^ and the United Nations Office for Disaster Risk Reduction UNDRR,^5^ disaster risk is defined as “the likelihood over a specified time period of severe alterations in the normal functioning of a community or a society due to hazardous physical events interacting with vulnerable social conditions, leading to widespread adverse human, material, economic, or environmental effects that require immediate emergency response to satisfy critical human needs and that may require external support for recovery”.^4^ From natural and technical sciences perspective, risk can be quantified by a deterministic risk equation (*1*).^6^

(Equation 1) $$R_{i,j}=f\left(p_{j},p_{i,j},A_{i},v_{i,j}\right)$$

Where $R_{i,j}$ is the risk dependent on object i and scenario j, $p_{j}$ is the probability of defined scenario j, $p_{i,j}$ is the probability of exposure of object i to scenario j, $A_{i}$ is the value of object i (the value at risk affected by scenario j), and $v_{i,j}$ is the vulnerability of object i in dependence on scenario j. An overview on differences of flood risk assessment considering different spatial scales is provided by de Moel et al.^3^

The flood risk analysis provides an important part for an overall risk governance (see in the study by Klinke and Renn^7^ for a broad overview including also sociopolitical perspectives) and especially for risk management. The aim of flood risk management is to reduce risk to an acceptable level for the relevant society via prevention, mitigation, preparedness, and response measures. It also includes managing residual risks and preventing new or increasing risk.^5^ The basis for understanding past, current and/or future risk in a flooding system and for selecting appropriate risk management measures is a pre-event risk assessment that considers the characteristics of the hazard, the exposure of elements at risk, and the vulnerability. Herein, the flooding system is defined as physical and human systems that “influence or are influenced by flooding”.^8^

However, flood risk is not static but changes over time.^6^^,^^9^^,^^10^^,^^11^^,^^12^^,^^13^ Flood events and risk are strongly related to climate change and this will raise further challenges for the global community.^14^ Moreover, land-use change, human interventions, and socio-economic developments all influence exposure and vulnerability, thus changes of all risk components have led and will lead to the evolution of flood risk.^15^^,^^16^^,^^17^ Consequently, the evolution of flood risk can be highly dynamic and complex.^18^^,^^19^^,^^20^ The complex properties lead to the emergence of systemic risks, which are characterized by interactions and transboundary effects in the scope of consequences,^21^^,^^22^ and need to be better understood and addressed in whole systems approaches.^5^^,^^23^^,^^24^ Approaches of risk analysis that address the evolution of risk in a comprehensive way and also take into account the interactions and feedback between hazards and the more societal risk components are scarce.^25^^,^^26^ Moreover, the future dynamics of drivers influencing hazard, exposure, and vulnerability, and consequently flood risk evolution is fraught with a high degree of uncertainty.

Understanding flood risk and the evolution of flood risk under global change conditions is therefore essential for sustainable decision-making in disaster risk reduction. This was already addressed in the Sendai Framework Priorities and targets by “focusing on monitoring, assessing, and understanding disaster risk” (Priority 1), as well as for “strengthening the mechanisms for monitoring and assessment of disaster risks” in the context of risk governance (Priority 2).^27^ However, monitoring tools mainly observe hazard and provide information on early warning as part of Priority 4 (Enhancing disaster preparedness for effective response and to “Build Back Better” in recovery, rehabilitation, and reconstruction)^27^ but not changing risk. Additionally, monitoring tools address the need for developing national and international platforms to monitor trends and patterns of disaster losses and impacts to properly capture any progress toward reducing losses.^5^^,^^28^ Other monitoring tools in relation to risk and risk reduction integrated in the Sendai Monitor (loss data and impacts, strategies for risk reduction) show little progress.^29^

Moreover, new assessment tools are needed to enable adaptive flood risk management to address the changing and systemic risks.^30^ However, focusing on understanding disaster risk and the implementation of adaptive flood risk management requires a flood risk monitoring that screens critical developments of hazard, exposure, and vulnerability in a comprehensive way and warns the decision makers when a critical point in flood risk evolution will be approached. In the context of reducing flood impacts on European societies, the EU Floods Directive 2007/60/EC^31^ provides guidelines to European Member States on flood risk assessment and management. The Flood Directive (FD) comprises three planning steps, (i) the preliminary assessment of flood risk and the identification of Areas of Potential Significant Flood Risk (APSFR), (ii) the establishment of flood hazard maps and flood risk maps, and (iii) the flood risk management plans to reduce the risks. These steps are to be repeated, reviewed, and, if necessary updated every six years. In 2022, most member states started the third cycle (2022–2027).^32^ However, the focus of the FD is not on the monitoring of risks—despite acknowledging the aspects of dynamic risk by the repeated assessment—but to assess risk to find adequate risk reduction measures. Moreover, the member states implemented very different approaches to address the FD^33^^,^^34^^,^^35^ and updated and changed the applied approaches from the first to third cycle which limits to observe the risk evolution. Yet, a scientific basis for risk monitoring serving for a proactive adaptive flood risk management is still missing.

Based on the highlighted challenges of the analysis and evolution of flood risk and the still existing limitation to address risk monitoring for an adaptive flood risk management, this study provides in a first step a review of local and regional flood risk evolution studies and in a second step an outline for a flood risk monitoring approach. We focus on pluvial and fluvial floods.

In the review part, we aim to present a synthesis of research on the evolution of flood risk through a systematic review of peer-reviewed articles to deepen the understanding of flood risk evolution. We analyzed the different approaches for addressing flood risk evolution, the dynamical risk components considered and the contribution of these publications on risk evolution for developing principles of flood risk monitoring. In the second part, we provide a critical perspective on flood risk evolution and distil the basic steps toward risk monitoring. We understand risk monitoring in this study as the systematic detection of risk evolution by periodically measuring the factors that influence the risk components hazard, exposure and vulnerability, modeling the risk components, and combining them to quantify risk. We aim to contribute with the iterative framework to support a better understanding of systemic risk and adaptive flood risk management.^21^

## Systematic review: Flood risk evolution

The following chapter includes the method description, an overview and classification of the selected publications, and the results and synthesis of the systematic review. The results and syntheses of publications are organized into sections according to whether they analyzed an evolution in all three risk components (*H* *=* *hazard*, *E* *=* *exposure*, *V* *=* *vulnerability*), in two risk components *(H*, *E*, or *H*, *V*, or *E*, *V*), or in one risk component *(H* or *E* or *V)*. The sections are sub-divided into publications analyzing an evolution in the past, future, or a comparison of past and future. Each sub-section answers the questions (a) what were the main objectives of the studies, (b) with which methods and factors was flood risk evolution and important drivers analyzed, and (c) what are the main conclusions regarding flood risk evolution and drivers.

### Method

**Figure 1:**
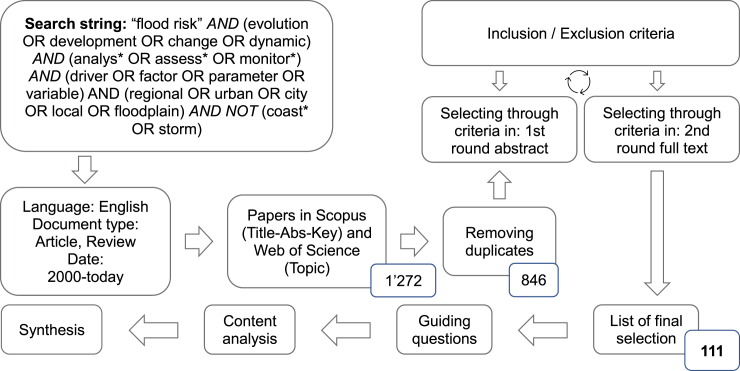
Experimental procedure systematic review

In order to elaborate principles of flood risk monitoring, we conducted a systematic search, review, and synthesis of peer-reviewed literature about flood risk evolution. This systematic review follows formal methodological steps outlined in Berrang-Ford et al.,^36^ Khan et al.,^37^ and Liberati^38^ to add transparency and reproducibility to the review process. The literature was searched with Scopus and Web of Science (last update: 19.04.2024) by using a search string (Figure 1) generated in an iterative process. Literature related to the research questions was screened to receive a set of search terms. After selecting search terms, various combinations were tested to obtain a search string that returned literature that met the eligibility criteria. Various Boolean operators were used to broaden and narrow the results. We searched within the article title, abstract and keywords fields in the Scopus database and in the topic field in Web of Science database. Additional search criteria were the language (English) and document type (article, review). The time of publication was limited to the period from 2000 until the date of the last search in Scopus and Web of Science (19.04.2024). After the removal of duplicates, 846 papers remained for the first selection. The retrieved titles and abstracts were screened according to pre-defined inclusion and exclusion criteria (Table 1). The second selection was done by reading the whole paper and screening according to pre-defined inclusion and exclusion criteria (Table 1). At the end, 111 papers were classified as relevant for the content analysis and synthesis. Undoubtedly, relevant publications may be missing in our literature search due to the varying use of terms in their titles, keywords or abstracts. Nevertheless, our results are robust in terms of flood risk evolution studies revealed.

**Table 1:** Inclusion- and exclusion criteria systematic review

Inclusion criteria	Exclusion criteria
Flood risk evolution analysis (Hazard, Exposure, Vulnerability, single or in combination) (past, present, future)	Model performance or uncertainty analysis
Identification or analysis of drivers of changing flood risk	Susceptibility-, hazard-, inundation-, risk mapping
Interaction and feedback between drivers	Resilience/Adaptation
Spatiotemporal dynamics	Evaluation of mitigation strategies/recovery/event management
	Evaluation of water pollution, quality/quantity
	Event/precipitation monitoring

After the final selection, we analyzed the literature (*n* = 111) following a set of guiding questions.

(1) How is flood risk conceptualized in the studies?
(2) What is the geographical region of the case studies?
(3) What is the temporal scale of risk analysis?
(4) Which factors are used for risk evolution analysis?
(5) Which methods are used to analyze flood risk evolution?
(6) What are the main outcomes from analyzing flood risk evolution?

The coding and content analysis of the studies was conducted in MAXQDA, a software for qualitative data and text analysis, following the guiding questions. After coding and content analysis, the studies were grouped according to the risk component(s) in which they analyzed an evolution and in what time span they analyzed an evolution of flood risk. Finally, the grouped literature was integrated in an excel-sheet to assemble the results of the content analysis and to synthesize the results. The selection, coding and content analysis of the literature was assessed by the first author of the manuscript in an iterative process with consultations and crosschecking of the co-authors.

### General overview and classification of analyzed publications

From total 111 papers selected for the review, 101 were research articles and ten were review articles. The earliest paper was published in 2003 and most of the articles were published in 2023. Case studies were realized around the world, with most located in Asia and Europe. In Table 2, the studies included for the systematic review (without review articles) are categorized by the applied risk definition (colored background), the risk components used to analyze an evolution (rows) and the time referred to by the risk analysis (columns). The studies analyzed the flood risk evolution under different risk conceptualizations, but how risk was defined and conceptualized was not always clearly presented in the studies. Moreover, 65% of the studies lacked a clear definition of risk (blue background in Table 2). For those studies we categorized the definition of risk according to their implicit descriptions of risk conceptualization. Although all studies mentioned flood risk, only 36% of the studies included all three risk components in their analysis. Flood risk, hazard, exposure, or vulnerability evolution was analyzed for either past or future or as a comparison of past, present, and future periods (columns). Most of the studies analyzed risk evolution in the past (52%) (first column of Table 2). 55% of the studies analyzed the evolution of one risk component. By combining more than one risk component, evolution was not always included in every risk component. 22 papers combined the evolution of all three risk components in the risk analysis.

**Table 2:** Categorization of reviewed studies

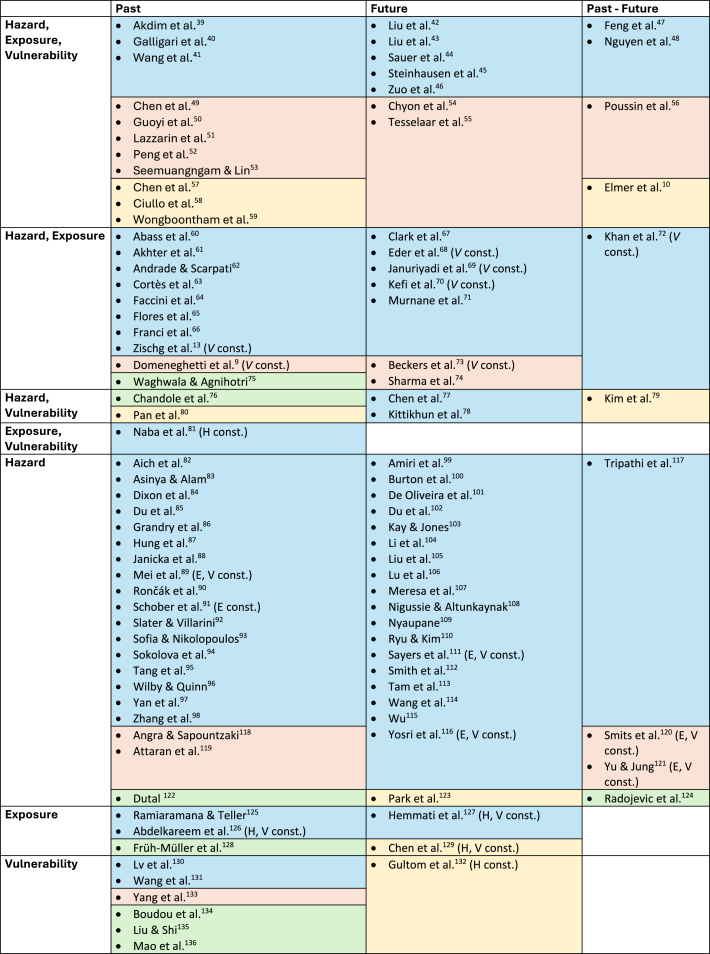

The studies were categorized according to three criteria: (a) the period for which the risk evolution was analyzed (past, future, past-future), (b) the integration of evolving risk components (*H=hazard, E=exposure, V=vulnerability*) and (c) the applied risk definition visualized by the colored background. The background color shows: blue = no risk definition, red = H x E x V, green = H x V, yellow = other. The italic letters in brackets indicate studies that hold one or two of the three risk components (*H, E, V*) constant over the analyzed period and considered the others as dynamically changing.

The selection of factors or the choice of model to analyze flood risk evolution varies across the analyzed studies depending on the risk conceptualization, research questions of the study, spatiotemporal scale of analyses, and data availability. Consequently, the comparison and synthesis between individual studies is quite limited. In the following sections, we present the summarized outcomes of the content analysis with a focus on the main objectives, and diversity of methods, key factors and results of flood risk evolution analyses.^9^^,^^10^^,^^13^^,^^39^^,^^40^^,^^41^^,^^42^^,^^43^^,^^44^^,^^45^^,^^46^^,^^47^^,^^48^^,^^49^^,^^50^^,^^51^^,^^52^^,^^53^^,^^54^^,^^55^^,^^56^^,^^57^^,^^58^^,^^59^^,^^60^^,^^61^^,^^62^^,^^63^^,^^64^^,^^65^^,^^66^^,^^67^^,^^68^^,^^69^^,^^70^^,^^71^^,^^72^^,^^73^^,^^74^^,^^75^^,^^76^^,^^77^^,^^78^^,^^79^^,^^80^^,^^81^^,^^82^^,^^83^^,^^84^^,^^85^^,^^86^^,^^87^^,^^88^^,^^89^^,^^90^^,^^91^^,^^92^^,^^93^^,^^94^^,^^95^^,^^96^^,^^97^^,^^98^^,^^99^^,^^100^^,^^101^^,^^102^^,^^103^^,^^104^^,^^105^^,^^106^^,^^107^^,^^108^^,^^109^^,^^110^^,^^111^^,^^112^^,^^113^^,^^114^^,^^115^^,^^116^^,^^117^^,^^118^^,^^119^^,^^120^^,^^121^^,^^122^^,^^123^^,^^124^^,^^125^^,^^126^^,^^127^^,^^128^^,^^129^^,^^130^^,^^131^^,^^132^^,^^133^^,^^134^^,^^135^^,^^136^

### Hazard, exposure, vulnerability evolution

22 of 111 studies analyzed flood risk evolution with evolving factors describing the hazard, exposure, and vulnerability component.

#### Evolution in the past

Studies that integrated an evolution of all three risk components into the risk analyses had varying research foci. The most common denominator is the evaluation and analysis of past conditions of the flooding system to learn about the evolution of this system. The gained knowledge is a key to a flood risk evolution analysis to compare different risk situations. The main objectives were to learn from past risk evolution for future decisions in flood risk management,^39^ to compare risk evolution in different regions to detect hotspots^41^^,^^57^ and flood management performance,^52^ to compare various risk situations with flood loss^49^^,^^58^ or to consider the impact of urbanization and settlement dynamics on risk evolution.^40^^,^^50^^,^^51^^,^^53^^,^^59^

The approaches selected for the analyses of flood risk under historic conditions were qualitative and quantitative data-, index-, and/or model-based analyses. After the collection of data for the state-describing variables for different periods, they were used to analyze inherent evolutions, to compare different states with occurred flood events or other evolutions of state-describing variables,^39^ or they were modified so that they can be used as input for index- or model-based evaluations.^40^^,^^41^^,^^49^^,^^50^^,^^51^^,^^52^^,^^53^^,^^57^^,^^58^^,^^59^

Index-based analyses described the risk components through several indicators (see for a general overview in the study by Sahani et al.^137^). Data for hazard evolution analyses were mainly precipitation time series (as proxy) and catchment characteristics. The amount of flood season precipitation,^57^ the amount of monthly precipitation, monthly maximum precipitation,^49^ the average annual precipitation,^50^ or maximum 3-day continuous precipitation^52^ were used as indicators for precipitation time series. As catchment characteristics, for example, changes in wetlands or vegetation cover rate,^49^ changes in normalized difference vegetation index (NDVI)^52^ or distance to river^50^ were selected as indicators. The indicators used for exposure analyses were, for example, population density, road density, economic density, built-up area, and/or the amount of gross regional or domestic product.^49^^,^^50^^,^^52^^,^^57^ Indicators for social vulnerability were, for example, the age of the population, the proportion of rural residents to the overall population,^49^^,^^52^ and/or coping capacities (e.g., municipal flood control investment per unit area).^57^ Indicators to assess the economic vulnerability were built-up density and proportion of farmland.^52^ Indicators were chosen *a priori* or selected via statistical analyses from a set of indicators to estimate their relevance. Finally, the identified indicators were aggregated in a risk index through weighting or statistical models. Risk evolution was detected by comparison of the resulting flood risk of several periods.

The model-based analyses integrated several descriptive variables of risk components into one stylized model^58^ or coupled data analyses with a hydraulic model.^40^^,^^51^ The selection of data for hazard evolution analyses depended on whether the study relies on flood-related variables (e.g., flood magnitudes, flooded area, high-water levels) or starts with the analysis of source variables of a flood event (e.g., precipitation or flood discharge) to model the flood-related variables with hydraulic models. Data for exposure evolution analyses were gross domestic product and population density^58^ or data for buildings.^40^^,^^51^ Data for vulnerability evolution analyses were proxies for flood protection levels, social memory of floods and relation between flood water levels and relative damage^58^ or structural characteristics of buildings.^40^ Lazzarin et al.^51^ integrated the vulnerability of residential buildings by using vulnerability functions. Ciullo et al.^58^ integrated time-invariant parameters and time-varying variables describing the three risk components into the dynamic model to detect risk evolution. Galligari et al.^40^ conducted several individual analyses of risk components and combined the knowledge gained to make statements about the risk evolution. Lazzarin et al.^51^ coupled a damage model to the hydrodynamic model to analyze the flood risk evolution.

Almost all analyzed studies came to the same result: the main reason for flood risk evolution was exposure evolution. Within each of the studies, the risk evolution in regions was compared and varying rising/decreasing flood risk detected. The study of Galligari et al.^40^ showed that built-up area and/or the number of buildings expanded but building density decreased. Regarding the vulnerability evolution, an increase in rooftop improvements was detected as adaptation strategy. Thus, related variables had an amplifying or dampening effect on the overall flood risk evolution. For example, the increase in building re-enforcement protected against heavy rainfall but not in case of increased runoff and water table rise.^40^ On the one hand, building consolidation decreased vulnerability of buildings against heavy rainfall, but, on the other, it increased vulnerability against a flood event. The study of Peng et al.^52^ showed a trend in first increasing and then decreasing risk for Beijing and an overall decreasing trend for Munich from 2000 to 2020. They stated that the reason for the differences lies in the urbanization process and the situation for flood risk mitigation.^52^ Lazzarin et al.^51^ showed in their case study an increase of 85% in expected damage from 1983 to 2021. Akdim et al.^39^ and Chen et al.^57^ stated that the relations in human-natural systems and factors describing flood risk evolution are multiple and complex.

#### Evolution in the future

Seven studies^42^^,^^43^^,^^44^^,^^45^^,^^46^^,^^54^^,^^55^ analyzed the expected future evolution of risk. The main objective of these studies was to analyze the impacts of climate-, socio-economic-, and land-use change on flood risk for different spatiotemporal scales. The evolution of risk was analyzed by a combination of data selection-, statistical-, index-, and/or model-based approaches. In contrast to the studies that analyzed evolution in the past, scenario-based simulations and statistical techniques were used to project and predict future changes in flood risk related factors.

Future hazard related factors were selected from available datasets,^46^ modified through modeled land-use change scenarios^42^^,^^43^ and/or modeled with a hydrodynamic model while integrating climate change and/or flood protection scenarios.^45^^,^^54^^,^^55^ Data for index-based hazard evolution analysis were, for example, precipitation time series (maximum three-day precipitation and number of days with daily rainfall above 50 mm) and catchment characteristics (digital elevation model, slope, topographic wetness index, distance to river, and runoff coefficient).^42^^,^^43^ In these examples, the runoff coefficient as indicator for hazard evolution was modified according to the new land-use types. Outputs from hydrological-, and hydraulic modeling (e.g., flood duration, flood depth, and flood extent) were modified as indicators (e.g., in the study by Chyon et al.^54^) or used to calculate statistics (e.g., changes in flood frequency and magnitude)^45^ and impacts (e.g., expected annual damage).^45^^,^^55^ Indicators for exposure and vulnerability evolution analysis were, for example, the total population per unit area and total general budget revenue per unit area. In this example,^42^ the changes in population and general budget revenue per unit area were spatialized with a multiple regression model. The results were combined with a weighted average method to work out flood risk maps for each year under each scenario (e.g., in the study by Liu et al.^42^). Steinhausen et al.^45^ used several methods to calculate population, GDP and wealth-to-income ratio for exposure evolution analysis. Vulnerability evolution was integrated by private precautionary measures, previous flood experience, building footprint area, and building type.^45^ Tesselaar et al.^55^ utilized population growth as input for exposure evolution analysis and depth-damage curves for vulnerability analysis.

The results showed an increase or decrease in flood risk depending on the time, region, and selected scenarios. Overall, future climate change will lead to an increase in flood risk but the dominant driver is socioeconomic change (e.g., in the study by Steinhausen et al.^45^). For example, Liu et al.^42^ stated that an increase comes mainly with removed vegetative surface, raw lands replaced by impervious area, and increased exposure with urban growth. Zuo et al.^46^ detected as well an increase in size and proportion of high flood risk areas, with population density and gross domestic product density as the most important drivers. Another driver of increasing flood risk, analyzed by Tesselaar et al.,^55^ is the offset of dis-amenities of floods by insurances. A stabilized flood risk was detected through a balance between socio-economic development and ecological protection measures.^42^^,^^43^ Additionally, Steinhausen et al.^45^ stated that improved private precautionary measures would reduce flood risk on average by 15%. Without these measures, fluvial flood risk can increase seven-, to 10-fold until the end of the century.^45^

#### Evolution from past to future

The studies analyzing risk under past and future conditions compared different periods to identify and estimate risk evolution. The focus was on quantifying risk in terms of expected annual damage to buildings and/or attributing risk changes to single drivers (climate change, land-use change, and change in building values).^10^^,^^47^^,^^48^^,^^56^

The approach selected for the analyses of flood risk evolution under historic and future conditions was a combination of data selection, model-, index-, and scenario-based analyses. Climate, hydrological, and hydraulic models were used to estimate damage and risk. For the quantification of flood hazard, discharge measurements and discharge projections provided the input for inundation modeling. Hazard probabilities were calculated with extreme value statistics of observed and predicted discharge data. To analyze the impact of climate change on hazard, climate change scenarios based on the IPCC emissions scenarios were used.^14^ Nguyen et al.^48^ calculated a flood hazard index by integrating flood depth, velocity and susceptibility from a machine learning and hydrodynamic model to create a flood hazard map. Exposure was quantified by analyzing land-use data from satellite images,^10^ land-use maps,^48^^,^^56^ and land parcel information.^47^ Future projections of land-use change were analyzed with the Land Use Scanner model and based on the IPCC emissions scenario.^10^^,^^56^ Additionally, Nguyen et al.^48^ integrated population density, poverty level, number of women, number of schools, and agricultural area as indicators from statistical offices for the analysis of exposure and vulnerability evolution. Future changes in population density and vulnerability indicators were collected from planning reports.^48^ In the other studies, structural vulnerability is represented by stage-damage functions^10^^,^^56^ or depth-damage fragility curves^47^ as relation between inundation height, land-use and building values. Changes in building values were considered by using time-adjusted reconstruction costs^10^ and published price indices.^47^ Additionally, Poussin et al.^56^ integrated risk mitigation factors into the stage-damage functions to analyze the effects of adaptation strategies on the damage and risk. Finally, different scenarios with related damage calculations were compared to detect the most relevant drivers of flood risk change. Nguyen et al.^48^ combined flood hazard, exposure, and vulnerability indicators with Geographical Information System (GIS) methodologies to construct flood risk maps and evaluate changes in flood risk areas.

The results showed that climate change, land-use change, and asset value developments affect flood risk in varying degrees. Almost all studies concluded that climate variations have an important impact on changes in flood risk but they were not the dominant driver, at least for the time period under study.^10^^,^^47^^,^^56^ Exposure evolution in form of land-use change was increasing under several scenarios and stated as the dominant driver.^10^^,^^47^^,^^56^ The asset value developments appeared to be a minor driver of flood risk evolution.^10^ The results of the overall risk estimations showed varying increasing/decreasing rates of the flood risk (represented by the expected annual damage) depending on the analyzed years (intervals) and scenarios. For example, Elmer et al.^10^ analyzed in their maximum land-use scenario a decrease of 30% in risk from 1990 to 2020 while considering effective building values. Whereas Feng et al.^47^ stated that the risk raised by 30% from 2001 to 2011 due to a combination of socioeconomic developments and climate conditions. Poussin et al.^56^ suggested that land-use and climate changes might increase annual flood risk by up to 185% by 2030 compared with 2000. With a focus on assessing and comparing flood risk areas, Nguyen et al.^48^ concluded that areas with high and very high flood hazard, exposure, vulnerability, and risk increased, while areas of low risk decreased due to a combination of climate change, land use change, population growth, and socio-economic growth.

### Hazard, exposure evolution

A large proportion of studies (18 of 111) analyzed flood risk evolution only with evolving factors describing the hazard and exposure component. Nevertheless, in some studies, the vulnerability component was integrated as a constant variable over time to calculate flood risk.

#### Evolution in the past

As stated in the previous chapter, the most common denominator of studies analyzing past risk evolution was the identification of past conditions of the flooding system to learn about the evolution and compare different risk situations. Even though this chapter deals with studies that do not considered an evolution in vulnerability, they had the same common denominator. The detailed objectives were not the same, as the studies had varying research foci. In detail, they examined factors that play an important role in flood dynamics^60^^,^^62^^,^^64^^,^^75^ and detected changes in factors describing the hazard and exposure component.^66^ Further, one study aimed to analyze and link changes in floodplain population dynamics with flood-related variables.^61^ Four studies focused on studying the evolution in flood risk, including hazard, exposure, and vulnerability (constant), how it has been affected by different changes and on detecting the main drivers of flood risk evolution.^9^^,^^13^^,^^63^^,^^65^

The approaches selected to analyze historic conditions of factors to determine past risk evolution were qualitative and/or quantitative data analyses, as well as index-based-, and/or model-based analyses. A comparison with observed flood events and/or discharge data served to evaluate which precipitation events caused a flood event and whether they showed a statistically significant change.^62^^,^^63^^,^^64^ Further, with this analysis, it was analyzed whether flood events were explainable through changes in climate or if other reasons were responsible for changed impacts of flood events. Land-use/cover analysis was used to examine changes in land surface and correlated change in runoff processes.^63^ In addition, the studies looked at the historical evolution of land-use in terms of urban area to detect changes in exposure due to urban sprawl.^64^^,^^66^ A more detailed study of exposure evolution in form of dynamics in floodplain population was conducted by Akhter et al.^61^ By analyzing floodplain population growth rate and the proportion of floodplain population compared with flood-related variables, they analyzed the impact of floods on dynamics in floodplain population.^61^

The index-based study^75^ calculated an urbanization and flood risk index to evaluate the impact of urbanization on two flood events. Data for hazard analysis were the inundation area, flood depth and discharge of these flood events. Data for exposure analysis was the urban area. The urbanization index was calculated as percentage of the urban area from the total study area. Finally, flood risk was analyzed with an average flood depth index and an urban submergence index.

Modeling studies analyzed the evolution in single risk components and then combined the results into a risk analysis. Data for hazard analysis were precipitation data as input for rainfall-runoff modeling to calculate various flood-related variables (discharge, water level),^65^ observed discharge data to statistically analyze trends^9^ and derived hydrographs to simulate floods.^13^ The hydrographs were scaled to various peak discharges and delineated to a certain occurrence probability.^13^ Further, flood-related variables (inundation extents and flow depths) were modeled with a hydrodynamic model.^13^ Data for exposure analysis were land-use maps to evaluate the expansion of urban and residential area in a flood-prone area.^9^ Further, population data were an important element for exposure analysis to analyze the number of people living in flood-prone areas.^9^ In a more general study of exposure, changes in land-use categories were analyzed through satellite image classification and overlaid with a certain flood extent.^65^ Finally, hazard and exposure analyses were combined into a risk evolution calculation to gain basic information for flood risk management and to determine which drivers were responsible for flood risk evolution.^9^^,^^13^

Factors describing climate-, land-use-, and population dynamics were the most important to analyze risk evolution. Nevertheless, the results of the studies showed that the evolution of these factors was highly variable depending on the selected location (e.g., Po river [Italy], Lujan river [Buenos Aires], metropolitan area of Barcelona [Spain], rural central Gonja district [Ghana]). For example, Abass et al.,^60^ Cortès et al.,^63^ Domeneghetti et al.^9^ and Flores et al.^65^ stated that they found no evidence that past floods were climate change-induced because of a lack of significant changes in precipitation or discharge time series. Andrade and Scarpati^62^ and Faccini et al.,^64^ however, stated that risk increased due to changes in precipitation patterns. The effects of changes in land-use and population growth on flood risk evolution also varied between studies. Domeneghetti et al.^9^ showed that flood risk (expected damage) doubled since 1954, mainly due to settlement growth. The review of the papers revealed that the dynamics in the flooding system cannot just be ascribed to the evolution of one single driving factor. For example, Akhter et al.^61^ stated that even if they detected a correlation between population dynamics and the influence of structural and non-structural measures, these dynamics cannot only be attributed to mitigation measures. Zischg et al.^13^ showed moreover that the construction of levees, and the effect of unintended river incision, decreased flood risk whereas settlement growth increased flood risk.

#### Evolution in the future

Studies analyzing future flood risk evolution focused on the analysis of impacts from climate change, urban development and/or management strategies on hazard and/or exposure evolution.^71^^,^^74^ Some studies quantified risk evolution in terms of calculating damage resulting from changes in the flooding system.^68^^,^^69^^,^^70^^,^^73^ Therefore, the main objective of these studies was to analyze changes in the hazard and exposure component under future scenarios and combine these evolutions into a risk evolution analysis.

The approach selected for analyses of future risk evolution due to changes in climate, socio-economic development, and risk management strategies was scenario-based modeling. Hazard evolution was analyzed with climate, hydrological and hydraulic models. The impact of climate change on hazard evolution was integrated in the analyses either through a change in precipitation depending on global climate models (GCMs) and representative concentration pathways (RCP) scenarios,^69^^,^^70^^,^^71^^,^^74^ an increase in peak discharge of 30%^73^ or a 10% addition to the flood hydrographs.^68^ In addition, one study analyzed the impact of land-use change and land subsidence on flood hazard evolution using a combined RCP and shared socio-economic pathway (SSP) scenario^69^ and one study analyzed the impact of planned flood protection measures on flood hazard.^68^ Finally, changes in flooded areas and water depths were modeled for the hazard evolution analyses. For example, future exposure maps (settlement areas) were generated in terms of future demand in land-use (population forecast and household size trend) and the location of new settlement areas under a current trend and a dense urban development scenario.^73^ Eder et al.^68^ based the projections of settlement development areas on information from policy documents and land-use plans. Another approach to predict future land-use change was the analysis of two previous land-use maps as input for a transition model to predict future land-use.^70^ On a coarser level, the SSP scenarios were used to develop future exposure data (e.g., GDP and population).^71^ The vulnerability component of risk was integrated as a constant variable for risk calculations. Finally, the risk evolution was calculated with the combination of inundation depths from the hazard evolution analyses, exposure from the exposure evolution analyses and related stage-, or depth-damage functions.

Results showed that hazard and exposure parameters will increase or decrease^68^^,^^73^ depending on the selected scenarios and combination of scenarios. For example, Beckers et al.^73^ stated that in a wet climate scenario the peak discharge would increase by 30% for the time horizon 2071–2100 while a dry climate scenario will lead to a slight decrease in the peak discharge. The degree to which climate change and land-use change will influence the risk evolution depends also on the selected scenarios.^73^ For example, Kefi et al.^70^ found that the potential damage will increase in one catchment due to climate change and in another the main driver of change in potential damage is the change in built-up areas. Overall, depending on the region, the analysis of direct damage under future scenarios showed an increase by 80% and 212%, respectively.^70^ Beckers et al.^73^ calculated for a wet climate scenario a relative increase in flood damage of 630% in 2100 with a 3–8 times higher influence of climate than the effect of land use change. Januriyadi et al.,^69^ in turn, found that future climate change and urban development will lead to an increase in flood risk (expected annual damage costs) by 322%–402% by 2050. In contrast, Eder et al.^68^ showed that the potential damage to buildings and land could decrease by up to 75% under future scenarios (flood protection measures, settlement development, and climate change).

#### Evolution from past to future

Only one study compared past and future conditions of a flooding system to analyze flood risk evolution. The objective of this study was to analyze the damage of a past flood event under a future urban development scenario.^72^

The approach selected for this analysis was a combination of observed data collection from an occurred flood event in past, a hydraulic model to reconstruct the flood event, a stochastic land-use/cover change model to project urban development scenarios and a damage assessment model. Data for hazard analysis were precipitation inputs, hydrological inflows, drainage control structures, cross sections of channels, and a digital elevation model. The future urban development scenario was used as input in the hydraulic model (affecting the hazard evolution) as well as in the damage assessment model (affecting the exposure evolution). Vulnerability, as structural vulnerability, was integrated as constant variable with associated depth-damage curves for residential and commercial properties.

They concluded that flood damage can increase significantly due to urban growth (up to 800%), with an impact on the hazard as well as the exposure component of risk.^72^ This is an example of a driver of change that influences two risk components, namely hazard and exposure.

### Hazard, vulnerability evolution

A very-small proportion of studies (5 of 111) analyzed flood risk evolution with evolving factors only describing the hazard and vulnerability component.

#### Evolution in the past

Past evolution of flood risk with changes in factors describing the hazard and vulnerability components was examined by two studies.^76^^,^^80^ The aim of these studies was to analyze spatiotemporal flood risk evolution and investigate drivers of flood risk evolution.

The studies used an index-based approach. Pan et al.^80^ used indicators describing the four influencing variables of hazard-causing factors (e.g., flood frequency), community vulnerability (e.g., proportion of population aged), protection works (e.g., plant cover), and systemic governance (e.g., proactive prevention). In contrast, Chandole et al.^76^ used spatially explicit criteria to calculate flood hazard (e.g., average annual rainfall, NDVI, distance from river, elevation) and flood vulnerability indicator (e.g., agricultural production, land use/land cover).

Pan et al.^80^ revealed that hazard related indicators had the largest weight and influence on flood risk. Chandole et al.^76^ stated that the high and very high risk areas increased, and the very low and moderate risk areas decreased from the Base scenario (before 2002) to the Advance scenario (after 2002).

#### Evolution in the future

Future evolution of risk with changes in factors describing the hazard and vulnerability components was examined by two studies.^77^^,^^78^ The aim of these studies was to analyze the impacts of urbanization, socio-economic development, land-use-, and/or climate change on future flood risk.

Future flood risk was analyzed through scenario-based modeling and index-based analyses. Chen et al.^77^ selected indices for the hazard and vulnerability component and modified a selection of indices with modeled scenarios. For the hazard component these were maximum 1-day rainfall amount, number of heavy rainfall days above 25 mm, areas in low-lying area, type of slope, runoff depth and distance to river.^77^ The digital elevation model, slope, and distance to river were kept as constant variables. Therefore, hazard evolution was analyzed by modifying precipitation data with regional climate models (RCMs) under selected RCP scenarios and by modifying the runoff depth under future urbanization scenarios from an urban growth model.^77^ Indices selected for the vulnerability component were the gross domestic product density and the population density.^77^ Vulnerability evolution was analyzed by modifying these two indices according to selected SSP scenarios.^77^ Finally, the hazard and vulnerability indicators were aggregated in a risk index through weighting and displayed as flood risk on maps.^77^ In contrast, Kittikhun et al.^78^ analyzed hazard evolution with modeled land-use change according to actual and planned land-use and integrating these changes into a rainfall-runoff model to model flood hydrographs and inundation areas. The vulnerability evolution was analyzed with the flood risk index composed of sub-indices describing exposure, susceptibility and resilience and modified with results from the modeled land-use changes.^78^ Finally, both results were combined to compare the flood risk index results under actual and planned land-use.^78^

The results showed that flood risk will increase in most parts of the studied area with differences between the selected climate/development scenarios.^77^ Kittikhun et al.^78^ concluded that flood risk will increase if no land-use planning aimed at risk reduction is applied.

#### Evolution from past to future

One study analyzed the comparison of past and future flood risk with evolving hazard and vulnerability component. It aimed at analyzing the risk situation before and after the completion of four major river restoration projects.^79^

The risk in the past and the future was analyzed by using an index-based approach. The index combined eight indicators describing the hazard component and ten indicators describing the socioeconomic vulnerability component.^79^ Past hazard was described with historical precipitation data and future hazard was analyzed with future climate change simulation data.^79^ The evolution of socio-economic vulnerability was analyzed with changes to six indicators.^79^ In addition, an expert-based weighting of indicators was applied.^79^

The results showed that the risk index increased after the river restoration project and would increase due to climate change.^79^ The study concluded that the risk reduction due to river restoration will be leveled off where floods are expected to increase due to climate changes.^79^

### Exposure, vulnerability evolution

One study analyzed flood risk evolution with evolving factors describing the exposure and vulnerability component. The hazard component was integrated as a constant variable over time.

#### Evolution in the past

The objective of this study was to quantify potentially exposed populations and investigate the relationship between poverty and flood exposure.^81^

They used GIS tools and remote sensing data to map populations affected by floods (exposure) and calculated a poverty index (vulnerability). While taking population and poverty values of different years, they assessed the evolution in exposure and vulnerability.

The results showed an increase in exposed people on a national scale but a decrease on a regional scale. The poverty index decreased over the years. Further, the results confirmed the relationship between floods, exposure, and poverty.

### Hazard evolution

A large proportion of studies (43 of 111) analyzed evolving factors describing the hazard component. In some studies, the exposure and/or vulnerability component was integrated as a constant variable over time to calculate flood risk.

#### Evolution in the past

Hazard evolution in the past is mainly analyzed out of an interest in reasons for changed hazard-related factors (flood magnitude, flood extent, water depth, etc.). The main objectives were to detect changes in the flooding system to examine their impacts on hazard-related factors and processes. The main drivers were climate trends, land-use change, and morphological changes. In comparison to the studies presented earlier, the level of detail is higher in the studies analyzing only the evolution in one of three risk components.

The approaches selected to detect changes in hazard-related factors from changes in the flooding system were the same as in the risk analyses studies and consisted of (statistical) data analyses, modeling and/or index analyses. (Statistical) data analyses were used to detect trends in observed and/or modeled precipitation, temperature, discharge and/or flood data and make them clearly visible.^82^^,^^84^^,^^86^^,^^93^^,^^97^^,^^118^ Yan et al.^97^ used a variety of environmental proxy reconstructions to examine how climatic and land-use changes affected floods. The main drivers of changes in hazard-related factors^92^ and their correlations^93^ were assessed by statistical models. In addition, statistical approaches were used to predict the evolution of magnitude and frequency of flood events.^83^^,^^85^^,^^90^^,^^91^^,^^96^ Flood-related variables with different settings were simulated with modeling approaches to evaluate the impact of climate-, land-use-, or morphological change on hazard evolution. To this end, observed or experimental climate, land-use/cover and morphological data were integrated into the models and several model runs were conducted under various settings to analyze the impact on flood-related variables.^82^^,^^83^^,^^84^^,^^88^^,^^90^^,^^91^^,^^95^^,^^119^ Information about land-use/cover change was integrated into the simulation models by means of land-use maps for representing different time periods.^77^^,^^82^^,^^98^ The analyzed land-use/cover categories vary across studies. For example, Aich et al.^82^ analyzed the changes in crop, pasture, savannah, water, and rock land-use cover. Dixon et al.^84^ and Sokolova et al.^94^ analyzed the impact of changes in forests, and Slater and Villarini^92^ used the harvested acreage of corn and soybean to represent agricultural practices. Rončák et al.^90^ used an experimental approach to analyze the impact of land-use/cover change on flood hazard. To do this, they created land-use scenarios and integrated these scenarios in a rainfall-runoff model.^90^ Channel or catchment morphological changes were also integrated in the hazard evolution analyses. For example, Asinya and Alam^83^ modeled and analyzed the effect of various synthetic channel morphological conditions (river width, bed elevation, etc.) on flood-related variables (flood inundation, flood frequency). Dixon et al.^84^ analyzed the effect of engineered logjams on channel morphology and thus flood-related variables (flood discharge). A detailed analysis of connections between longitudinal variability in river conveyance, flows, sediment connectivity and flood changes was conducted by Sofia and Nikolopoulos.^93^ Mei et al.^89^ used an index-based approach with constant exposure and vulnerability indices. Therefore, the change in flood risk was obtained only with changing hazard indices (changing rainfall regimes).^89^ Dutal et al.^122^ used an index-based approach to identify flood hazard zones in two different years. With the overlay of flood hazard maps and two land-use maps, they revealed the effects of urbanization on flood hazard zones.^122^ Hung et al.^87^ applied a machine learning and remote sensing approach to assess continuous inundation susceptibility and the effects of climate change.

The results showed that climate-, land-use/cover-, river channel morphology change and river restoration projects were important drivers. Nevertheless, the drivers of detected changes were a mix of factors and the determination of causes of changes is highly complex due to the interaction between factors intervening in flood processes.^86^^,^^93^

#### Evolution in the future

The main objective of future hazard evolution analyses was to examine the impact of climate change scenarios on flood-related variables and processes, as well as analyzing uncertainties. Yet three studies analyzed the impact of future land-use change scenarios on hazard evolution.

The relevant approaches were (statistical) data analyses, scenario-based modeling, and/or index-based analysis. The focus of these studies was on using future climate projections from global and RCMs under RCPs as input for hydrological and hydraulic modeling. The generated flow time series were used to analyze changes. For example, Kay and Jones,^103^ Lu et al.,^106^ Meresa et al.^107^ and Wang et al.^114^ extracted the annual maxima from the flow time series to detect changes in flood frequency. In addition, they built land-use change and urban development scenarios based on past observations as well as future plans from municipalities and/or assumptions of socio-economic developments and then integrated them in hydrological and hydraulic modeling.^100^^,^^101^^,^^104^^,^^108^ Sayers et al.^111^ generated flood events under climate change and used a statistical empirical copula to generate a large number of unseen events to calculate future fluvial flood risk. Park et al.^123^ used four indicators (precipitation data) to analyze the impact of climate change on flood hazard without hydrological or hydraulic modeling. Du et al.^102^ analyzed the trend in future extreme precipitation events under climate change with intensity-area-duration methodology. Yosri et al.^116^ used a deep learning approach to predict future flood risk under climate change.

Results showed that future climate change will contribute to an increase in flood-related variables and proxies (e.g., extreme runoff, flood magnitude, inundation extent).^104^^,^^106^^,^^107^^,^^109^^,^^110^^,^^111^^,^^112^^,^^115^^,^^123^ Nevertheless, as stated by Kay and Jones,^103^ Liu et al.,^105^ Ryu and Kim,^110^ and Wang et al.,^114^ changes in discharge and flood frequency and magnitude varied in spatial distribution and statistical significance depending on catchment and selected RCMs. Additionally, Kay and Jones^103^ and Liu et al.^105^ stated that the results are associated with several uncertainties and hydrological changes are often non-linear. While analyzing urban growth, land-use and climate change scenarios, and integrating these scenarios in hydrological and hydraulic models, drivers of change in flood-related variables were tested.^100^^,^^101^^,^^104^ Results showed that determining and modeling future changes and analyzing their impacts on hazard evolution provides useful information for flood risk management.^100^^,^^104^ The analysis of the impact of urbanization under three land-use policy scenarios on flood inundation indicated that unrestricted urbanization will lead to an increase in inundated land.^108^

#### Evolution from past to future

Hazard evolution analysis in past and future was conducted by four studies. They aimed to analyze the impact of land-use change and/or climate change on hazard evolution. Radojevic et al.,^124^ Smits et al.^120^ and Yu and Jung^121^ analyzed flood risk evolution in the past and future with changes in the hazard component and constant exposure and/or vulnerability. Tripathi et al.^117^ analyzed and compared the impact of future climate change and urban development on a past flood event.

The hazard evolution was analyzed with statistical methods and hydrological/hydraulic modeling or index-based analysis. Statistical tests were used to analyze the evolution of flood frequency and severity in the past.^124^ Changes in flood regimes were analyzed by integrating land-use data from different periods into the models.^124^ Tripathi et al.^117^ integrated climate change (increase in precipitation) and urban development (increase in impervious surface) into a hydrological model to analyze the impact on the peak flow of a past flood event. Smits et al.^120^ and Yu and Jung^121^ utilized modified precipitation indicators under different climate change scenarios to assess flood hazard evolution.

Results indicated that land-use change from peri-urban development had a different effect on flood regimes depending on the selected spatial scale of analysis.^124^ By comparing the outcomes of a past flood event with future conditions, Tripathi et al.^117^ detected that an increase in urban areas will lead to a higher impact of the flooding. In addition, the longer inundation time and higher peak flow will lead to higher damage.^117^ Results of the index-based analyses showed different increasing and decreasing hazard (risk) areas with a high spatial variability.^120^^,^^121^ They concluded that the analyses of risk areas and cause analysis of flood risks served a useful tool for decision makers to find strategies for climate change adaptation.^120^^,^^121^

### Exposure evolution

A very-small proportion of studies (5 of 111) analyzed evolving factors describing the exposure component of flood risk. In some studies, the hazard and/or vulnerability component was integrated as a constant variable over time to calculate flood risk.

#### Evolution in the past

The main objectives of these studies were to analyze the long-term historic development of settlements and population in terms of flood exposure.^125^^,^^126^^,^^128^

Exposure evolution was examined by (statistical) data analysis. The spatial data of settlement areas were overlapped with measures such as distance to flooding zones, extent of floodplain and topographic variables to analyze exposure evolution.^128^ In addition to settlement data for exposure analysis, Ramiaramanana and Teller^125^ used demographic data to analyze the evolution in number of inhabitants. Combining the percentage of population and built-up areas in flood prone areas served to detect socio-economic drivers and challenges.^125^ Abdelkareem and Mansour^126^ used remote sensing analysis to detect changes in vegetation and infrastructure.

The results showed that exposure strongly increased in the past. Früh-Müller et al.^128^ stated that the total built-up area within the flooding zone of the studied area increased almost 5-fold. Ramiaramanana and Teller^125^ also found that over the selected time period the built-up area increased yearly by 6.1%.

#### Evolution in the future

Exposure evolution in future was analyzed by Hemmati et al.^127^ and Chen et al.^129^ The aim of Hemmati et al.^127^ was to analyze the interaction between urbanization and flood risk to enhance the knowledge about non-structural mitigation measures. The main objective of Chen et al.^129^ was to analyze the effect of economic change on flash flood risk.

An urban growth-, hazard-, risk analysis-, and policy implementation model was used to examine flood risk under various urban development scenarios.^127^ While keeping the hazard and vulnerability component constant, the exposure component in terms of urban growth evolved over time under several policy scenarios. Chen et al.^129^ used a hydrological/hydrodynamic model for hazard assessment, asset value spatialization for exposure analysis and a flash flood damage model for risk assessment. Five economic scenarios from the shared socioeconomic pathways (SSPs) were used to calculate exposure evolution.

Results showed that exposed people and assets might increase under current urban development plans, but that considering non-structural strategies can mitigate the consequences of floods.^127^ Chen et al.^129^ stated that the flash flood risk under economic change will increase by the end of the century by around 90% depending on the scenario selection.

### Vulnerability evolution

A very-small proportion of studies (7 of 111) analyzed evolving factors describing the vulnerability component of flood risk.

#### Evolution in the past

Vulnerability evolution analysis varied between studies depending on the selected conceptualization. The studies assigned to this category mainly aim for a quantitative analysis of impacts of land-use change, socioeconomic evolution and/or disaster risk management on the vulnerability evolution.

Vulnerability evolution was mostly analyzed by evaluating the exposure (elements at risk) and the susceptibility of the elements at risk. Therefore, data of exposure and susceptibility were evaluated and combined with data-, index-, and model-based analysis to examine vulnerability evolution. Data and indices selected to analyze vulnerability evolution are built-up area, building-use type, population living per building, population density, regional GDP and land-use type/categories in comparison with historical flood data.

Past vulnerability evolution is uncertain and complex.^134^ The results showed an increasing or decreasing trend depending on local characteristics and the assessed periods.^131^^,^^133^^,^^134^^,^^136^ For example, Yang et al.^133^ concluded in their case study that human and economic vulnerability was steadily declining from 2000 to 2020. Lv et al.^130^ stated that reasons for changing vulnerability in the past were an increase in building land, inflation and mismatch between urban growth and mitigation measures.

#### Evolution in the future

The aim of Gultom et al.^132^ was to analyze vulnerability in present and future to determine the evolution of vulnerability or resilience.

To this aim, they analyzed the evacuation route efficiency by using space syntax methods and sheltering capacity determined by data and simple equations. The calculation was repeated with projected population data to predict future changes.

The analysis and comparison of flood vulnerability showed an increase in resilience due to the implementation of a ring road having a positive effect on the evacuation routes.

## From flood risk evolution to flood risk monitoring

The analyzed studies presented great variety and diversity in the approaches. This hampers comparability, but the review allows drawing conclusions on flood risk monitoring and distilling the principles of risk monitoring.

As seen from the previous chapters, flood risk evolves through a combination of dynamically changing factors in the flooding system. These factors, represented by data or proxy data that change over time, quantify the evolution of the risk components (*H,E,V*). The combination of the changing risk components in a risk analysis allows analyzing flood risk evolution.

The presented publications analyzed flood risk evolution by repeating flood risk analysis in regular (e.g., 1- or 10-year) periods^10^^,^^57^ or for selected years of interest.^42^^,^^47^ In general, all spatial scales are represented, from very small-scale studies,^73^ to catchment-wide,^9^ and national^61^ studies. The selection of periods and spatial scales depends on the risk factors assumed as changing dynamically over space and time, on the availability and resolution of data, and on methodology.

Flood risk evolution analyses, integrating the evolution of hazard, exposure and vulnerability, use comparative (statistical) data-, index-, model-, and scenario-based analysis approaches. These approaches are used either as stand-alone risk calculation methods or in combination. Comparative (statistical) data analyses are useful to detect the diversity of changing factors in a flooding system.^39^ Nevertheless, changing variables can only be analyzed for the past and the quality is determined by the amount of data that is available at comparable levels of accuracy over a long period. The quantification of flood risk according to the narrow definition of this term is missing in data-and index-based approaches. Index-based analyses are useful to compare risk between spatial units and to identify risk-hotspots.^49^^,^^57^ Repeated surveys of the risk index can prove the effectiveness of risk management strategies.^49^ Nevertheless, although the parameter selection is flexible, the results are method-dependent and not transferable to other regions. Hence, comparability of flood risk evolution over several regions is limited. Furthermore, risk is quantified on an aggregated level of a region and detailed statements about local impacts of flood hazards on exposure and vulnerability are not possible with index-based approaches. With model-based analyses, flood hazard evolution can be quantified, and exposure and vulnerability can be derived from the simulations and geospatial overlay analyses. The outcomes of model-based risk evolution studies are comparable across regions. Model-based analyses allow analyzing the effects of change in an individual risk factor (e.g., settlement growth) on the overall risk evolution, as well as analyzing the combined effects of all changing risk factors. The studies that consider the change of more than one risk component show that the evolution of the risk factors can have self-reinforcing (cumulative) effects on overall risk evolution or even cancel each other out and keep overall risk nearly constant. Only a model-based approach allows for disentangling the individual effects of changing risk factors on the overall risk evolution and thus to identify the most relevant driver of change in a flooding system. A disadvantage of model-based analyses is the amount and resolution of data and computational power needed. Scenario-based analyses can enhance risk evolution analyses by integrating possible influences of climate and socio-economic changes.

**Figure 2:**
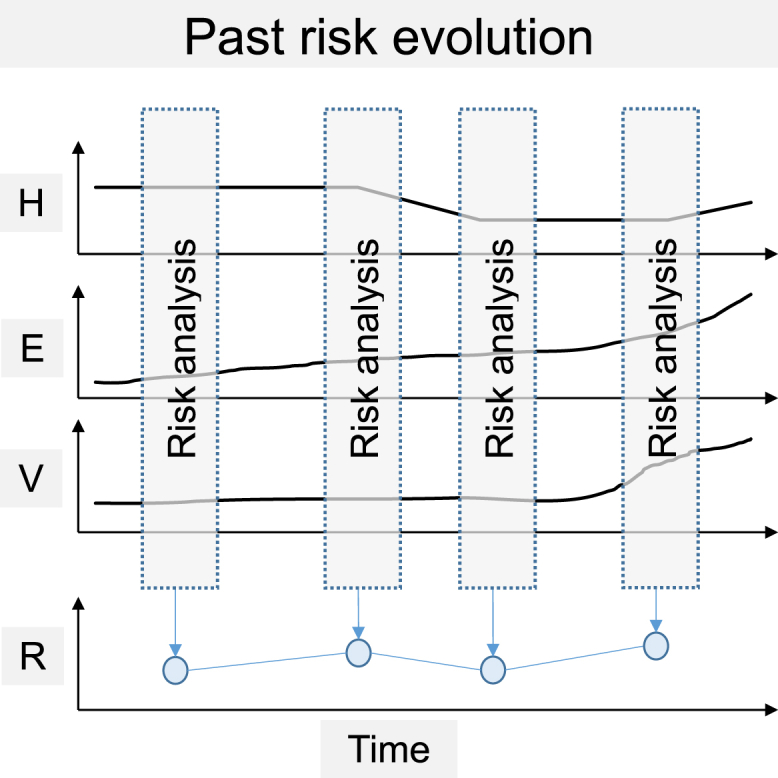
Illustration of past risk evolution The risk analysis combines the three risk components (*H=hazard*, *E=exposure*, *V=vulnerability*). Each component (*H*, *E*, *V*) has its own evolution over time. The repeated risk analysis over several time steps detects an increase or decrease of risk in past, and therefore risk evolution.

The repeated analyses of risk in the past allow the detection of flood risk evolution (Figure 2). Further, they enable the detection of drivers of change and the system dynamics leading to an increase or decrease of risk. In addition, the analyses of past risk evolution enable the detection of events and management strategies that had or will have (delayed) effects on the risk system. While analyzing past evolution, it is possible to ascertain past developments leading to limitations for future evolutions (legacy effect). Nevertheless, to evaluate risk evolution in the past, difficulties arise with data availability, data completeness, data accuracy, and scales over periods. Consequently, the comparability of the risk analysis outcomes over multiple periods is not always given and the availability bias must be taken into account. In addition, the risk analyses are prone to uncertainties from the climatic/hydrological/hydraulic analyses as well as from exposure and vulnerability analyses.^138^

**Figure 3:**
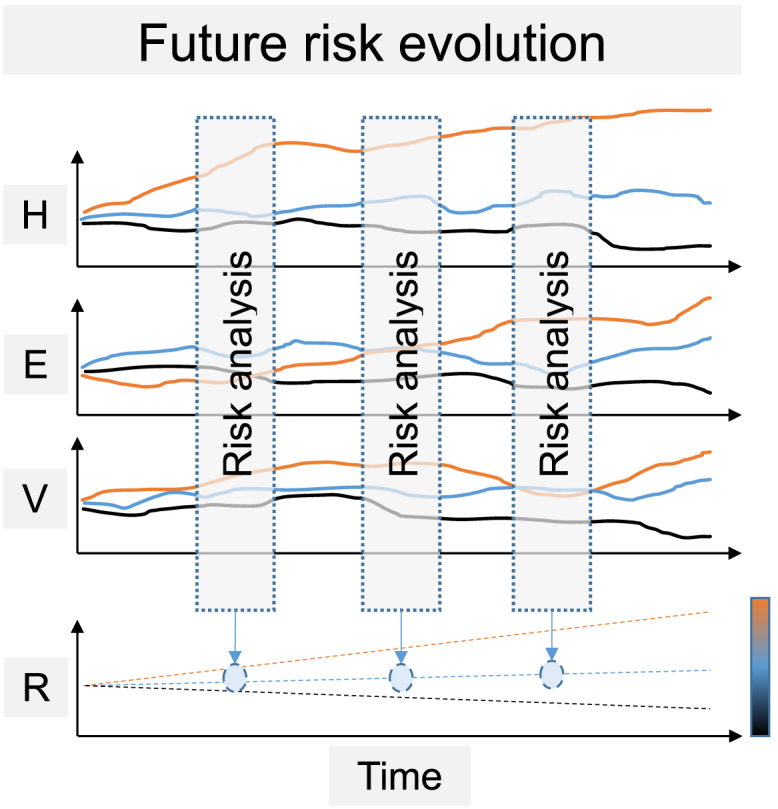
Illustration of future risk evolution The evolution of all three risk components (*H=hazard*, *E=* e*xposure*, *V=vulnerability*) in the future can be analyzed using several future scenarios. As in the past, the possible risk evolution can be projected while analyzing risk for several time steps. Comparing the periods reveals trends in risk evolution.

The analyses of future risk evolution allow identifying possible drivers of change and pathways of risk evolution (Figure 3). Future risk prediction enables the detection of critical thresholds that are important for adaptive flood risk management. However, future estimations of hazard, exposure, and vulnerability are based on scenarios and are therefore prone to uncertainties.^139^ It is therefore crucial to monitor, which of the underlying scenarios are becoming effective. Depending on the spatiotemporal scale of the case study, the risk increases or decreases due to different explanations. For example, Elmer et al.^10^ detected a decrease in risk for residential buildings from the years 1990–2000 that can be attributed to changes in flood hazard, and an increase in risk for residential buildings from the years 2000–2020 due to changes in exposure (urban sprawl).

## Flood risk monitoring

Although the review focused on flood risk evolution, the conclusions can be generalized and are valid for monitoring risk evolution from other natural hazards as well. The use and interpretation of the term monitoring varies between disciplines. The IPCC defines monitoring as “systematically identifying, characterizing, and assessing progress over time”.^4^ However, monitoring tools mainly observe the hazard and provide information on early warning^27^ or observe global and national patterns of disaster losses and impacts.^5^ The monitoring approaches listed previously capture only parts of the overall risk. However, risk results from the interactions between the risk components (*H*, *E*, *V*), consequently, risk itself cannot be monitored directly. Additionally, risk monitoring cannot be done by just monitoring a single data stream; it requires the combination of data and proxy data of the evolving risk factors and thus methods such as data mining, data modeling, data analyses, and data combination.

In conclusion, we define the term “risk monitoring” as the systematic detection of risk evolution by periodically (re)evaluating the factors influencing the risk components hazard, exposure, and vulnerability, modeling the risk components and combining them to quantify risk.

**Figure 4:**
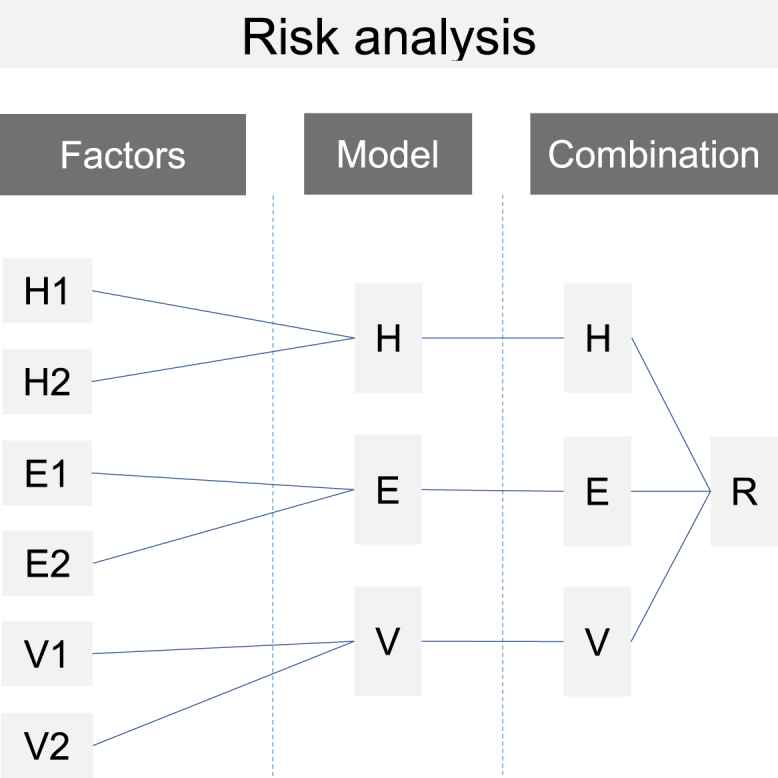
Principles of risk analysis Several monitored factors are used to model each of the risk components (*H=hazard, E=exposure, V=vulnerability*). The risk components are combined to quantify risk (*R*).

The most important component of a monitoring is the risk analysis framework (Figure 4). It describes how risk is quantified and how the risk factors describing the three risk components hazard, exposure, and vulnerability are determined. These factors need to be integrated in a model framework to calculate the three risk components. Modeling the risk factors is needed if the evolution of risk factors must be derived from proxy data that can be monitored quantitatively. After modeling and combination of data that represent the risk factors, risk can be analyzed and evaluated.

**Figure 5:**
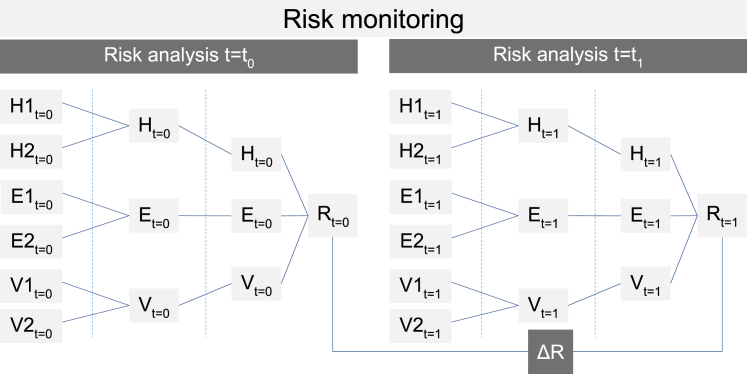
Principles of risk monitoring Systematic detection of risk evolution (*ΔR*) by periodically (t = t_0_, t = t_1_) measuring the factors influencing the risk components hazard (*H*), exposure (*E*), and vulnerability (*V*), modeling the risk components and combining them to quantify risk.

By repeated risk analysis, risk values can be compared over time and trends detected (Figure 5). After at least two repeated quantitative risk analyses, a risk monitoring reveals an additional dimension in risk analysis. It shows if risks in a certain place are increasing or decreasing. The trend can be quantified in a rate of change. This in turn allows estimating the period in which a certain risk threshold will be reached and when risk will not be societally acceptable anymore and risk mitigation measures are required. This informs decision-making in adaptive risk management in addition to knowing the current state of risks. Risk monitoring is a monitoring approach independent of the selected time step length between different time steps (e.g., every year or every ten years). This contrasts with other monitoring setups where selected state-describing variables are being monitored continuously (e.g., discharge). While risk monitoring is made on discrete time steps, monitoring changes in the risk components can in principle be a continuous monitoring of data streams.

### Opportunities and challenges

To elaborate flood risk monitoring, it is necessary to determine what risk monitoring can or should be used for. Essential objectives of flood risk monitoring are (a) to gain a better understanding of flood risk evolution, (b) to identify spatiotemporal variations of flood risk evolution, (c) to maintain the safety level of flood risk management, and (d) to detect possible drivers that lead to an increase or decrease in flood risk. This allows detecting legacy effects, rebound effects, time delays, and the effects of the implementation of flood risk management strategies. Following on from the target setting, it must be defined which actors will use the results for which target group and for which kind of decision-making. It must be defined in the beginning whether the risk is analyzed qualitatively, semi-quantitatively, or quantitatively. A strict quantification of risk evolution is possible only with model-based approaches. The objective and purpose of risk monitoring also determine the selection of data or factors to be examined.

This review summarizes the data and factors that change and can be monitored to analyze the evolution of risk. Hence, the review points out examples for the variability of potential risk monitoring setups and provides a starting point for the selection of methods and factors to be considered in the design of a risk monitoring concept.

The comparability of data over a long time period is one of the most important challenges. Once a risk monitoring concept is drafted, it must be guaranteed that the necessary data for periodically repeated risk analysis are continuously updated and expected to be available for the next decades. In addition, several sources of uncertainty exist in the risk analyses in general and, consequently, in risk monitoring as well. Examples are the definition of hazard scenarios, the process of modeling (input data, calibration etc.), the data quality, as well as uncertainties in the vulnerability analysis. The data availability is closely linked with the objectives of the risk monitoring. Given the diverse approaches to analyzing risk, the combination of approaches can serve to evaluate the spatiotemporal evolution of risk. Depending on the objective, varying levels of detail are necessary and useful. Another important aspect is the spatial delineation of the system. Different spatial reference systems must be used and combined, such as political units, hydrological catchments, or raster cells. While the risk components hazard and exposure are mostly used in a similar meaning across the reviewed publications, we found great diversity in the use and definition of the term “vulnerability”. How the evolution of this risk component is implemented in a risk monitoring concept must be carefully evaluated.

## Conclusion

In this study, flood risk evolution analysis studies were systematically reviewed. The review shows that there is no one size fits all applicable risks monitoring strategy. However, there is a variety of studies which analyzed flood risk evolution in the past and for the future and which served as a basis to elaborate principles of a flood risk monitoring. Further, the results of the review can help further researchers to find appropriate methodology and factors to analyze flood risk evolution and/or set up a national/regional or local flood risk monitoring concept.

The detailed monitoring of factors describing the risk components allows disentangling important changes in risk components that lead to an increase or decrease in risk. This disentangling means that management measures can be implemented specifically to address the main drivers of change.

Further research is needed in holistic risk analyses, including dynamics in hazard, exposure and vulnerability. The focus should be on a complex system perspective to analyze non-linearity, interactions between risk components, co-evolution in risk components, and feedback mechanisms.^19^ In addition, further research and implementation of monitoring studies is necessary for enabling adaptive flood risk management. The focus should be on data mining, validation, warranty of consistency, and modeling frameworks.

## Acknowledgments

The authors would like to thank Brigitte Scott for the language editing.

## Author contributions

Lead contact, N.R.; conceptualization, N.R., A.Z., M.K.; methodology, N.R.; data visualization, N.R.; writing-original draft, N.R.; writing-review and editing, A.Z. and M.K.; supervision, A.Z. and M.K.

## Declaration of interests

The authors declare no competing interests.
